# Trajectory patterns of behavioral and psychological symptoms of dementia (BPSD) in long‐term care facility residents in Japan and the United States

**DOI:** 10.1002/alz.086058

**Published:** 2025-01-09

**Authors:** Asuna Arai, Yuriko Katsumata

**Affiliations:** ^1^ Faculty of Medicine and Graduate School of Medicine, Hokkaido University, Sapporo, Hokkaido Japan; ^2^ College of Public Health, University of Kentucky, Lexington, KY USA; ^3^ Sanders‐Brown Center on Aging, University of Kentucky, Lexington, KY USA

## Abstract

**Background:**

Behavioral and psychological symptoms of people living with dementia (BPSD) are considered to reflect the person’s quality of life and are also risk factors for increased burden on caregivers. Although symptoms of BPSD are known to be diverse and variable, it is unclear whether they show similar longitudinal patterns in different countries. Therefore, this study aimed to clarify the trajectory patterns in BPSD and related factors using data from Japan and the US and compare the results of the two countries.

**Method:**

Using cohort study dataset that were followed up approximately annually in Japan and the US, we extracted data for four waves since 2015. In the US data drawn from the study at National Alzheimer’s Coordinating Center (NACC), long‐term care facilities included two categories such as the assisted living, adult family home, or boarding home, and skilled nursing facility, nursing homes or hospitals. In the Japanese data, long‐term care facilities were categorized into three types including adult group home for people with dementia, long‐term care health facility, and intensive care home for the elderly. A group‐based multi‐trajectory modeling was used to identify the longitudinal patterns of 12 BPSD symptoms over time. We also examined the characteristics of the participants belonging to each trajectory group.

**Result:**

The long‐term care facility residents used for analysis were 480 in Japan and 164 in the US. Three trajectory groups of BPSD were identified among the participants both in Japan and the US. When comparing between two countries, the trajectories of these groups showed similar patterns. Factors associated with the probability of group membership tended to differ by group and also by country.

**Conclusion:**

We identified three similar trajectory groups of BPSD in Japan and US long‐term care facility residents. It is suggested that the different backgrounds between countries did not have a great impact on the longitudinal pattern of multiple BPSD symptoms. It would be possible for Japan and the US to consider the BPSD preventive measures for residents in the long‐term care facilities in a common framework.

